# Ulcerative colitis and Crohn's disease: is *Mycobacterium avium *subspecies *paratuberculosis *the common villain?

**DOI:** 10.1186/1757-4749-2-21

**Published:** 2010-12-17

**Authors:** Ellen S Pierce

**Affiliations:** 1 Spokane Valley, Washington, USA

## Abstract

*Mycobacterium avium*, subspecies *paratuberculosis *(MAP) causes a chronic disease of the intestines in dairy cows and a wide range of other animals, including nonhuman primates, called Johne's ("Yo-knee's") disease. MAP has been consistently identified by a variety of techniques in humans with Crohn's disease. The research investigating the presence of MAP in patients with Crohn's disease has often identified MAP in the "negative" ulcerative colitis controls as well, suggesting that ulcerative colitis is also caused by MAP. Like other infectious diseases, dose, route of infection, age, sex and genes influence whether an individual infected with MAP develops ulcerative colitis or Crohn's disease. The apparently opposite role of smoking, increasing the risk of Crohn's disease while decreasing the risk of ulcerative colitis, is explained by a more careful review of the literature that reveals smoking causes an increase in both diseases but switches the phenotype from ulcerative colitis to Crohn's disease. MAP as the sole etiologic agent of both ulcerative colitis and Crohn's disease explains their common epidemiology, geographic distribution and familial and sporadic clusters, providing a unified hypothesis for the prevention and cure of the no longer "idiopathic" inflammatory bowel diseases.

## Introduction

Ulcerative colitis and Crohn's disease are chronic diseases of the gastrointestinal tract that together are usually referred to as idiopathic inflammatory bowel disease (IIBD). The word "idiopathic" means "of unknown cause," but the currently accepted theory of the cause of these diseases postulates a nebulous combination of immune dysfunction, "dysbiosis," [1] and otherwise normal constituents of the commensal extracellular luminal gut bacteria [2] leaking through an abnormally permeable mucosal layer [3] in genetically predisposed individuals [4].

The main alternative theory of Crohn's disease causation is infection with a facultative intracellular bacterium, *Mycobacterium avium*, subspecies *paratuberculosis *(MAP) [5-8]. MAP causes a chronic disease of the intestines [9,10] in a variety of animals [11-16], including nonhuman primates [17], that shares some histologic (i.e., microscopic) similarities to the changes found in Crohn's disease.

This commentary proposes that ulcerative colitis is also caused by MAP. The epidemiology, geographic distribution [18] and clusters (both sporadic and familial) of ulcerative colitis and Crohn's disease are consistent with MAP as the single etiologic agent of both diseases. Like other infectious diseases [19], a variety of factors including dose, route of infection, age, sex and genes influences whether an individual infected with MAP develops ulcerative colitis or Crohn's disease.

## Discussion

### Crohn's disease in unrelated individuals is an infectious disease; it is caused by a microorganism transmitted in water by the fecal-oral route

Let's begin in the middle of the story. What causes Crohn's disease, and how are ulcerative colitis and Crohn's disease related to one another? In 2001, Van Kruiningen and Freda [20] published their study of a "clustering of Crohn's disease in Mankato, Minnesota," that described 7 unrelated individuals with Crohn's disease in the city of Mankato, all members of the Mankato West High School class of 1980. The circumstances of the development of their Crohn's disease led the authors of this study to conclude that these cases of Crohn's disease were caused by

environmental transmission, transmission of a modestly contagious microbial agent via a fecal-oral route...related to bacterial surface water pollution...(Crohn's disease) may have been contracted via bathing or swimming in contaminated waters, via contamination of fresh vegetable crops, or via surface water contamination of wells during flooding [20].

Fecal-oral transmission means that a microorganism present in the feces of an animal contaminates the food or water that a person swallows. This one study concluded that Crohn's disease in unrelated individuals is an infectious disease; that is, it is caused by a microorganism, found in animal feces, that contaminates water.

### Crohn's disease in families, in genetically related individuals, is also an infectious disease

Genetically related individuals, families who have several members with Crohn's disease, also develop Crohn's disease because they are infected with the causative microorganism. In 1993, Van Kruiningen and colleagues published a "Study of Crohn's disease in Two French Families." [21] In the first family, the father developed Crohn's disease in 1970. In 1974, within several months of each other, 2 of their 4 children developed Crohn's disease. In 1982 and 1983, the other two children developed Crohn's disease. Finally, in 1988, the mother developed Crohn's disease. In the second family, 7 of the 11 children developed Crohn's disease, again in a pattern (first 4 of the children within 10 months of each other, then 6 years later another child, then 6 years after that, 2 more siblings) that led the authors of this study to conclude that "an enteric pathogen" caused these families to develop Crohn's disease:

The uniformity of ileal and cecal disease in family 1 is akin to that which might be expected had a uniform dose of an enteric pathogen been given to a genetically uniform group of experimental subjects, e.g., a litter of mice or piglets [21].

The conclusions that can be drawn from these two studies are that genetically unrelated people develop Crohn's disease because they have been infected with the causative microorganism, and genetically related people also develop Crohn's disease because they have been infected with the causative microorganism.

### Sporadic Crohn's disease, Crohn's disease in genetically unrelated individuals, is the same disease as familial Crohn's disease, Crohn's disease in genetically related individuals

We have thus far determined that Crohn's disease, whether in related or unrelated individuals, is an infectious disease, meaning that people develop Crohn's disease because they have been infected with the causative microorganism. But is sporadic Crohn's disease the same kind of disease as familial Crohn's disease? Is the infectious microorganism that causes unrelated people to develop Crohn's disease the same microorganism that causes families to develop Crohn's disease? Studies of families with Crohn's disease have concluded that there is no essential difference between the pathology of Crohn's disease in families with the disease and in unrelated individuals with the disease. Since there are no essential differences in the pathology of sporadic versus familial Crohn's disease [22-25], the organism that causes them must also be the same.

### How is ulcerative colitis related to Crohn's disease? The same infectious microorganism that causes Crohn's disease sometimes causes ulcerative colitis instead

The studies of sporadic and familial clusters of Crohn's disease conclude that Crohn's disease is caused by an infectious microorganism, and that, whether sporadic or familial, Crohn's disease is the same disease. How, then, is ulcerative colitis related to Crohn's disease? Let's return to those "two French families" [21], where a total of 13 individuals developed Crohn's disease. In the second of these families, in which 7 of the 11 children developed Crohn's disease, one affected child "presented initially with diffuse mucosal colitis and may have had ulcerative colitis as well as Crohn's disease." [21] The same "enteric pathogen," the same "infectious microorganism" that caused the 13 individuals in these two families to develop Crohn's disease caused one of those 13 individuals to also develop ulcerative colitis.

The same microorganism that causes the rare patient to develop *both *Crohn's disease and ulcerative colitis [26-29] usually causes some individuals to develop one disease *or *the other. Numerous studies of "mixed" families [30-34], where some individuals in a family develop ulcerative colitis and others develop Crohn's disease, are usually used to illustrate the idea that Crohn's disease and ulcerative colitis are genetic diseases, that some combination of genes causes both diseases. If, however, Crohn's disease is an infectious disease, these same studies of "mixed" families can just as easily illustrate the idea that if an infectious microorganism causes Crohn's disease, that same infectious microorganism sometimes causes ulcerative colitis instead [30-34].

If the same microorganism causes some genetically related individuals to get ulcerative colitis and others to get Crohn's disease, and if sporadic Crohn's disease (Crohn's disease in genetically unrelated individuals) is the same disease as familial Crohn's disease (Crohn's disease in genetically related individuals), then sometimes unrelated people will get ulcerative colitis rather than Crohn's disease when infected with the same microorganism. This is illustrated by the first of the two French families, where both the (genetically unrelated) mother and the father developed Crohn's disease [21], as well as the studies of unrelated spouses who both develop IBD: in some cases both spouses develop Crohn's disease [35-37], in some cases one spouse develops Crohn's disease and the other ulcerative colitis [38-40], and in some cases both spouses develop ulcerative colitis [38-41].

Unmarried and unrelated individuals living together also sometimes develop ulcerative colitis rather than Crohn's disease when infected with the same microorganism. In one of these nonfamilial or sporadic clusters, Aisenberg and Janowitz [42] describe 3 friends who shared living quarters in college. Several years after they left school, 2 of the friends developed Crohn's disease, and 1 developed ulcerative colitis.

The idea that ulcerative colitis and Crohn's disease have the same etiology is supported by consideration of the general epidemiology of these two disorders:

The significant linear regression between the incidence of Crohn's disease and ulcerative colitis from different countries and a similar relationship between the mortalities from both diseases could mean that the two diseases share a common factor in their etiology [43].

All studies (of the populations affected by IBD) thus far have failed to show that the population which develops Crohn's disease differs in any important feature from that which develops ulcerative colitis [44].

Crohn's disease and ulcerative colitis have similar demographic features. They...occur in the same places and at similar times. Where Crohn's disease is common, so is ulcerative colitis [45].

### Which specific infectious microorganism is found in patients with Crohn's disease ...and ulcerative colitis? MAP

MAP is an organism found in animal feces. It infects and causes clinical disease, called Johne's ('Yo-knees') disease, in a wide variety of animal species. Several meta-analyses and reviews of the literature have concluded that the MAP organism is consistently associated with Crohn's disease [46-48]:

...the MAP Crohn's disease phenomenon has fulfilled at least four (strength of association, consistency of effect, temporality and biological plausibility) of the six epidemiological causal criteria outlined by Hill...the current epidemiological evidence strongly supports the conjecture that Crohn's disease is caused by MAP [46].

There is an association between MAP and (Crohn's disease), across many sites, by many investigators, and controlling for a number of factors [48].

...our meta-analysis showed an association between MAP and Crohn's disease that is robust and specific [49].

The studies attempting to identify the MAP organism in Crohn's disease by a variety of techniques have often used ulcerative colitis as the "negative" control, under the assumption that MAP may cause Crohn's disease, but not ulcerative colitis [47,48]. When MAP is identified in *both *Crohn's disease and ulcerative colitis, researchers conclude that MAP causes *neither *disease. If, however, the current body of research is looked at from the point of view that MAP might cause ulcerative colitis *as well as *Crohn's disease, there is evidence to support this alternative position.

Like the organism that causes tuberculosis, *Mycobacterium tuberculosis*, that infects one-third of the world's population [50] as estimated by positive tuberculin skin testing [19], serum antibodies to various MAP antigens are found in a third of the population in countries with a high incidence of ulcerative colitis and Crohn's disease [51] and in patients with both diseases [51-53]. MAP can be identified by IS900 PCR (polymerase chain reaction) in the tissues [54-58] and cultured from the bloodstreams [59] of patients with ulcerative colitis as well as patients with Crohn's disease.

For a pathologist such as the author, the most convincing evidence of causation is direct visualization: can the organism be seen under the (light) microscope? The evidence from direct visualization is actually the strongest support for the idea that MAP causes ulcerative colitis as well as Crohn's disease: MAP can be directly visualized in patients with both diseases [60,61]. In the single study that directly visualized MAC (*Mycobacterium avium *complex) organisms (of which MAP is one) in almost 60% of Crohn's tissues using traditional acid fast stains [60], MAC organisms were also seen in 40% (2 of the 5) of the ulcerative colitis tissues. However, these researchers went so far as to reclassify their ulcerative colitis patients as having Crohn's disease, rather than concluding that MAC organisms can be directly visualized in *both *ulcerative colitis and Crohn's tissues:

...the observation of positive results in a few controls requires further clarification ...2 of the 5 control subjects with positive results had ileal disease but an assigned diagnosis of UC (ulcerative colitis); if these patients instead were classified as Crohn's disease, the Odds Ratio would increase from 8.6 to 17.1 [60].

If the data are accepted that MAP is present in both ulcerative colitis and Crohn's disease tissues, the conclusion can be drawn that MAP causes both diseases. Why, then, do some patients develop ulcerative colitis rather than Crohn's disease when infected with the same microorganism?

### When infected with MAP, why do some individuals develop ulcerative colitis rather than Crohn's disease? The usual influences of dose, route, age, sex and genes on the clinical expression of an infection

Like any other pathogenic (disease-causing) microorganism, a variety of factors influences the phenotype, the clinical expression of MAP infection; whether an individual develops one disease caused by MAP or another. Not everyone infected by a pathogenic microorganism develops a disease caused by that organism. "Infection" means the "multiplication of the organism in or on the host." [19] In contrast, "disease represents a clinically apparent response of the host to infection." [19] A review of the literature suggests that five factors influence whether an individual develops ulcerative colitis or Crohn's disease when infected with MAP:

1) The dose of the MAP organism, or how many organisms infect an individual. For a given age, a small dose of MAP causes ulcerative colitis, while a large dose of MAP causes Crohn's disease.

2) The route of infection. The literature suggests that routes of infection that increase the concentration of MAP increase the risk of developing Crohn's disease rather than ulcerative colitis. The two routes of infection that potentially concentrate MAP are aerosolized water from rivers and other bodies of water contaminated with MAP, and MAP present in hyperosmolar milk rather than hypoosmolar water.

3) The age at which an individual is infected. It takes a smaller dose of MAP to cause either ulcerative colitis or Crohn's disease in a child as compared to an adult.

4) Specific genes that control how an individual processes intracellular bacteria, and specifically mycobacteria. Individuals with these genes have a slighter greater tendency to develop Crohn's disease rather than ulcerative colitis, when infected with the MAP organism.

5) Whether an individual is male or female. Infant males and adult females seem to have a greater chance of developing Crohn's disease rather than ulcerative colitis when infected with MAP.

### The interactive influences of age and dose: when infected with a small dose of MAP, *adults *develop ulcerative colitis relatively soon after being infected, while *children *develop Crohn's disease after a long latency period

An examination of another family with both diseases suggests why some people get ulcerative colitis and others get Crohn's disease when infected with the same microorganism. This family had been living in a section of Germany with water that "was poorly controlled hygienically." [62] The mother, the adult, developed ulcerative colitis only 6 months after living in the area. Her two children developed Crohn's disease, one 4 years after living in the area and the other 5 years after.

This pattern of development, first of ulcerative colitis, then several years later of Crohn's disease, is illustrated occasionally at the individual level in patients who first develop ulcerative colitis, and then approximately 10 years later develop Crohn's disease [27]. More commonly, in high incidence countries, the incidence of ulcerative colitis first increases, followed several years later by Crohn's disease [63-65]. At the population level, immigrants from low to high prevalence IBD countries first develop ulcerative colitis [66], then again after approximately a decade begin developing Crohn's disease [67-70]. Adult and child immigrants from low to high prevalence IBD countries are infected at the same time, but the adults develop ulcerative colitis relatively soon after being infected, while the children develop Crohn's disease approximately 5 to 15 years after infection. The latency period between infection with MAP and the development of Crohn's disease is consistent with the literature on Johne's disease, where animals are infected as young animals but don't develop the clinical disease until they are adults [71].

As countries become westernized, meaning specifically the introduction of MAP-contaminated beef and dairy foods and the corresponding environmental pollution by MAP-infested beef and dairy cattle feces, their populations first develop ulcerative colitis and then again after a delay of several years develop Crohn's disease:

As societies become more "westernized" or industrialized...ulcerative colitis emerges, but the incidence of Crohn's disease remains low. Over time, Crohn's disease emerges, and its incidence ultimately matches that of ulcerative colitis [69].

The ratio of CD (Crohn's disease) to UC (ulcerative colitis) has changed over the past 50 years, with early reports in the mid-20^th ^century describing a predominance of UC cases in the United States and Western Europe. A gradual rise in CD cases in the 1960's through the 1990's resulted in CD incidence surpassing UC incidence in most westernized countries. Studies from Japan recapitulate the US experience, with a predominance of UC cases early and a rise in CD approximately 1 decade later [67].

### For a given age, infection with a small dose of MAP causes ulcerative colitis, while infection with a large dose of MAP causes Crohn's disease

At the microscopic level, a "small number" of organisms means 1 or 2 microorganisms within a single cell, requiring x1000 magnification to be seen under the light microscope [60], while a "large number" of organisms means a group of 3 or more organisms within a single cell, that can be identified at a magnification of x400 [72]. At the macroscopic level, however, a small number of organisms, a small "bacterial burden" or a small "dose" of MAP refers to numbers of organisms on the order of 10^7^, 10 million organisms or less, while a large bacterial burden refers to doses on the order of 10^8 ^or 100 million organisms or more.

In a recent study, Smith and colleagues [73] demonstrated that patients with Crohn's disease have trouble clearing large numbers of subcutaneously injected *Escherichia coli *organisms from their tissues:

Bacterial clearance was dramatically different between the two (HC or healthy control and CD or Crohn's disease) groups. In HC subjects, clearance rates were relatively stable between 10^5 ^and 3 × 10^7 ^organisms and actually increased at the highest dose, whereas in CD, although it was normal at 10^5 ^and 10^6^, there was a dramatic and highly significant drop in clearance at and above 10^7 ^bacteria [73].

These researchers concluded that Crohn's patients can clear small doses of bacteria from their tissues, but not large doses:

Most acute infections originate from a small number of inoculating organisms, which even the dampened immunity in CD appears able to control. Our findings of the relationship between bacterial dose and clearance are important in this respect because they demonstrate that CD patients can deal efficiently with small numbers of organisms in the tissues, but that the clearance mechanisms are overwhelmed by a large bolus of bacteria [73].

These researchers discuss the idea that the ileum and proximal colon are exposed to large numbers of luminal bacteria, specifically in the range of 10^8 ^in the ileum and 10^11 ^in the colon, and speculate that Crohn's patients might have trouble handling these massive doses of normal luminal bacteria [73]. However, an alternative explanation is that patients with Crohn's disease do not have trouble handling massive loads of normal luminal bacteria, but do have trouble handling large doses of MAP.

MAP is one of the subspecies of the *Mycobacterium avium *complex (MAC) group of microorganisms [74]. Normal water treatment processes such as filtration and chlorination amplify rather than eliminate MAC organisms by killing off their competitors [75,76]. In addition, MAC organisms grow on tap water pipes [77], in biofilms [78] and on plastic water bottles [79]. MAP has been identified in drinking water in the United States [80,81] and elsewhere [82]. It is estimated that MAC organisms may be present in drinking water on the level of up to 700,000 or 7 × 10^5 ^organisms per liter of water [77]. If an individual drinks 2 liters of water a day, it will take just over 2 months to ingest 10^8 ^or "massive numbers" of MAP organisms.

Once the level of MAP organisms in drinking water regularly reaches hundreds of thousands of organisms per liter, the chances of ingesting such large doses of MAP reaches a saturation level [43] and the rate of Crohn's disease and ulcerative colitis in a population stabilizes, with the chances of ingesting large doses of MAP generally being greater than ingesting small doses. Therefore, in populations with a high prevalence of IBD, there is usually a higher prevalence of Crohn's disease than ulcerative colitis [67,83-85].

As discussed above, when infected with a small dose of MAP, an adult develops ulcerative colitis relatively soon after infection, while a child develops Crohn's disease many years after infection. As the level of MAP organisms increases in the environment, including drinking water, a child has a greater chance of getting infected with a large dose. The author proposes that children infected with a large dose of MAP develop Crohn's disease with a shorter latency period between infection and clinical disease, including newborn infants [86].

In the study above [73], ulcerative colitis patients were able to clear large doses of *E. coli*, unlike the Crohn's patients. The author believes that ulcerative colitis patients have a more aggressive response to any dose of MAP, whether small or large, whereas Crohn's patients can handle small doses of MAP but not large ones. So patients develop ulcerative colitis even when infected with small doses of MAP.

### The two routes of MAP infection: manure and milk

MAP is excreted in an infected animal's feces and secreted in its milk. There are, therefore, 2 different routes of infecting humans with MAP: fecal-oral transmission via contaminated water, and consumption of contaminated milk or products made from contaminated milk. A careful examination of the literature reveals that routes of infection that result in concentrating MAP result in a greater likelihood of developing Crohn's disease rather than ulcerative colitis.

The first route of transmission of MAP to humans is fecal-oral transmission. "Fecal-oral spread is an important means of transmission of a variety of bacterial, viral and parasitic diseases...It may involve...finger-to-mouth spread, the use of night soil as fertilizer, or fecal contamination of food or water" [19]. The reported clusters of Crohn's disease associated with fecal-oral transmission have involved the use of manure as fertilizer [21,87] and possible fecal contamination of treated (tap) water [88], untreated (well) water [89] and surface water used for recreation [20]. Direct contact with dairy cattle feces has also been associated with an increased risk of ulcerative colitis in adult male farm workers [90].

MAP causes chronic intestinal disease in animals whether ingested, injected subcutaneously or intravenously, or inhaled [7], and aerosolization from possibly MAP-contaminated bodies of water, particularly rivers, is associated with an increased risk of Crohn's disease as compared to ulcerative colitis. There is a particularly high incidence of Crohn's disease in cities with rivers running through them, as exemplified by the author's home of Spokane, Washington, in the United States [91] and Cardiff, in the United Kingdom [85,92-94]. In Cardiff, the high-incidence Crohn's disease "wards" are adjacent to the river in a pattern exactly downwind of the prevailing wind pattern, in contrast to ulcerative colitis cases that are spread throughout the city's wards [94-96]. The author believes that the MAP organism is concentrated in the aerosolized water bubbles, and it is this concentration of MAP bacteria to large numbers by the aerosolization process [97] that results in Crohn's disease rather than ulcerative colitis in infected patients.

The second route of MAP transmission to humans is through contaminated milk. Despite the concerted study of the possible transmission of MAP to humans from pasteurized milk [98,99], the reported clusters of Crohn's disease suggest that it is unpasteurized or raw milk or milk products that carry the greater risk of infection. The author believes that the potentially large numbers of MAP in raw milk [21] and cheese [87] are the cause of the associated clusters of Crohn's disease; whereas the logarithmically smaller number of MAP organisms [100] in pasteurized milk [101] might trigger ulcerative colitis [102,103].

Finally, the literature suggests that MAP in milk is more invasive than MAP in water, because of the organism's passage through mammary gland epithelial cells and because of the hyperosmolar nature of milk [104]. The author speculates that the association between sugary cereals and an increased risk of Crohn's disease [105-107] and ulcerative colitis [108,109] may be a result of the increase in osmolarity of the milk caused by the sugar in the cereal, and decreasing the osmolarity of fluid in the digestive tract by carbohydrate-restricted diets [110,111] may be the cause of their reported efficacy in treating both Crohn's disease and ulcerative colitis.

### Crohn's disease and ulcerative colitis are not genetic diseases, but genetic polymorphisms related to the processing of intracellular bacteria, and specifically mycobacteria increase the risk of developing Crohn's disease rather than ulcerative colitis, when infected with MAP

Previous authors have suggested that ulcerative colitis and Crohn's disease have the same etiology, with Crohn's disease patients having more of the predisposing genes than ulcerative colitis patients:

Crohn's disease and ulcerative colitis may be the same disease with similar causes and with a quantitatively related genetic basis. Crohn's disease would be the result in people with a large concentration of genes, while fewer of the relevant genes would result in ulcerative colitis [112].

The accumulation of genetic polymorphisms associated with an increased risk of developing Crohn's disease as compared to ulcerative colitis supports this contention. The NOD2/CARD15 protein is an intracellular pattern recognition receptor that appears to not only recognize intracellular bacteria in general, but bacteria from the genus *Mycobacteriae*, including *M. leprae *[113,114] and MAP [115]. The ATG16L1 and IRGM proteins are involved in the process of autophagy, which is one of the ways cells process and eliminate intracellular bacteria [116]. The NRAMP (natural resistance associated macrophage protein) 1 gene [117] is associated with an increased risk of developing Johne's disease in sheep [118] as well as Crohn's disease [117], leprosy [119] and pulmonary MAC infections [120]. The NRAMP protein is expressed in reticuloendothelial cells [121], a possible location of the MAP organism in humans with Crohn's disease [122]. Individuals with one or more of these genetic polymorphisms, all of which make it slightly more difficult for individuals to process intracellular bacteria, have a slighter greater chance of developing Crohn's disease rather than ulcerative colitis, when infected with MAP.

Individuals with these genetic polymorphisms do not, however, develop Crohn's or ulcerative colitis in any significant numbers. Infection with MAP causes Crohn's disease and ulcerative colitis independently of having these polymorphisms. "In other words, regardless of whether you carry the mutant allele or not, *M. avium paratuberculosis *infection has the same tendency to produce CD" [123]. Depending on the country and region, these genetic polymorphisms are not associated with Crohn's disease [68,124].

### The influence of sex: boys develop Crohn's disease more easily than girls, while men develop ulcerative colitis and women develop Crohn's disease, when infected with MAP

It is commonly known that infant males are more prone to infectious diseases than infant females, probably due to weaker immune systems [125]. This weaker immune response might cause boys to develop Crohn's disease more often than ulcerative colitis [67,126-128] when infected with MAP, because they probably have a weaker immune response to the MAP organism. It has been postulated that Crohn's disease is not an autoimmune disease, a disease of excessive or over reactive immunity, but an immune deficiency disease [129]. Individuals with Crohn's disease have a less active response to the MAP organism, in contrast with individuals with ulcerative colitis, who have a more active response, as proposed above.

In contrast to children, adult females seem slightly but consistently more likely to develop Crohn's disease [64,130] and adult males to develop ulcerative colitis when infected with MAP. While purely speculative, this difference might be due to the differing effects of testosterone [131] versus estrogen [132] on blood and lymphatic vessels, to the greater effects of smoking on females than males [133], or to the increase in oral contraceptive use, with its known vascular complications [134].

### Smoking increases the risk of both ulcerative colitis and Crohn's disease, but nicotine has different effects on colonic versus small bowel inflammation

There are two commonly accepted beliefs about the relationship between smoking and Crohn's disease and ulcerative colitis: smoking increases the risk of Crohn's disease but protects an individual from developing ulcerative colitis, and smoking increases the severity of Crohn's disease, but decreases the severity of ulcerative colitis. If ulcerative colitis and Crohn's disease are both caused by MAP, how can these apparently dichotomous effects be explained? An excellent review article by Lakatos and colleagues [135] helps elucidate the reasons for the different effects of smoking on these diseases.

First, in children, smoking increases the risk of developing both ulcerative colitis and Crohn's disease. In a study of inflammatory bowel disease in Kentucky children [126], children who started smoking before 10 years of age were 7 times more likely to develop ulcerative colitis than nonsmokers, and over 3 times more likely to develop Crohn's disease than nonsmokers. If they started smoking before age 15, they were over three times more likely to develop both diseases. Smoking increased the risk of developing both Crohn's disease and ulcerative colitis, and in this one study increased the risk of ulcerative colitis (7 times greater risk) more than it increased the risk of Crohn's disease ('only' 3 times greater risk).

Secondly, the literature commonly cites the statistic that ulcerative colitis occurs in nonsmokers and ex-smokers, but again a careful examination reveals that smoking does not protect someone from developing ulcerative colitis; it causes them to develop Crohn's disease instead. Bridger and colleagues [136] demonstrated that in siblings, smokers develop Crohn's disease while nonsmokers develop ulcerative colitis. They write:

We have shown...that in some cases smoking can probably displace the phenotype of chronic IBD from UC to CD...The explanation of the apparent "protective" effect of smoking on sporadic UC may be that the form of IBD that develops in a proportion of smokers is not UC but CD [136].

Not only does smoking predispose a MAP-infected individual to develop Crohn's disease rather than ulcerative colitis, it favors the development of small bowel disease [137], due to the effect of nicotine on reducing colonic inflammation as discussed below.

The idea that smoking predisposes an individual infected with MAP to develop Crohn's disease rather than ulcerative colitis is supported by the high prevalence of ulcerative colitis in members of the Mormon Church, "where smoking is strongly discouraged" [135]. In a study of Mormons in Britain and Ireland, ulcerative colitis was five times more common in Mormons than in the general population [138] while the prevalence of Crohn's disease was the same. In comparative studies of Hindus and Muslims in the United Kingdom [68,139-141], Hindus (more likely to be nonsmokers) had high rates of ulcerative colitis compared to Muslims (more likely to be smokers); not smoking switched the phenotype from Crohn's disease to ulcerative colitis. The lack of smoking did not protect nonsmoking populations from developing ulcerative colitis but instead caused them to have higher rates of ulcerative colitis compared to populations who smoked, keeping the overall prevalence of IBD the same but inverting the usual ulcerative colitis/Crohn's disease ratio.

But smokers with ulcerative colitis do have a more benign clinical course than nonsmokers. If MAP causes ulcerative colitis, what in the smoke is good for ulcerative colitis? It is the nicotine in the smoke that helps *both *colonic Crohn's disease and ulcerative colitis. First, nicotine reduces tone and muscular activity in the colon; it slows the colon down [142]. Secondly, nicotine increases small bowel inflammation but decreases colonic inflammation [143,144]. Transdermal nicotine patches help both ulcerative colitis patients and patients with colonic Crohn's disease [145].

So the superficial paradox of the effects of smoking on Crohn's disease and ulcerative colitis is rather easily explained. Smoking increases the risk of both diseases, and increases the risk that the clinical expression of MAP infection will be Crohn's disease rather than ulcerative colitis.

### Conclusion: hope for the prevention and cure of the no longer idiopathic inflammatory bowel diseases

It is likely that MAP is the cause of a range of human gastrointestinal diseases including irritable bowel syndrome [146,147] as well as ulcerative colitis and Crohn's disease. MAP has also been investigated in the pathogenesis of other so-called autoimmune diseases including sarcoidosis [148,149], type-1 diabetes [150-152] and Hashimoto's thyroiditis [153]. If we accept that both ulcerative colitis and Crohn's disease are caused by a specific infectious microorganism, MAP, a long overdue transformation will take place in the prevention and treatment of these diseases. A rapid, focused effort to determine and implement the most efficacious treatments of already infected individuals [110,111,154-158], and methods of preventing initial infection by vaccination [159] and the effective removal of MAP organisms from drinking water [160] will become top priorities. We can end the "public health tragedy" [161] of Crohn's disease, and ulcerative colitis, in our lifetimes.

*Dedicated to Cyrus E. Rubin, M.D*.

## Competing interests

The author declares that no competing interests exist.

## Acknowledgements and Funding

My research would not be possible without the assistance of Gail Leong, Sandy Keno and Dr. Dodie Ruzicki at the Sacred Heart Medical Center Health Sciences Library in Spokane, Washington, as well as the other libraries that participate in the FreeShare library group within the DOCLINE National Network of Libraries of Medicine.

No funding was received for the preparation of this commentary.
